# *In Vitro* Assessment of Magnetic Liposomal Paclitaxel Nanoparticles as a Potential Carrier for the Treatment of Ovarian Cancer

**DOI:** 10.34172/apb.2021.039

**Published:** 2020-08-05

**Authors:** Sara Yousefi Aldashi, Zahra Saffari, Hasan Ebrahimi Shahmabadi, Azim Akbarzadeh

**Affiliations:** ^1^Islamic Azad University Faculty of Technical and Engineering, Science and Research Branch, Tehran, Iran.; ^2^Department of Pilot Nanobiotechnology, Pasteur Institute of Iran, Tehran, Iran.; ^3^Department of Microbiology, School of Medicine, Rafsanjan University of Medical Sciences, Rafsanjan, Iran.

**Keywords:** Cytotoxicity, Drug delivery, Magnetic liposome, Nanoparticles, Ovarian cancer, Paclitaxel

## Abstract

***Purpose:*** This study aimed to evaluate the role of magnetic liposome nanoparticles (ML NPs) as a carrier for paclitaxel (PTX) for the treatment of ovarian cancer *in vitro*.

***Methods:*** Magnetic NPs (MNPs) were synthesized by chemical co-precipitation method. The resulting NPs were characterized in terms of size, size distribution, zeta potential, drug encapsulation efficiency (EE), drug release pattern, and cytotoxicity effects.

***Results:*** The size and zeta potential of PTX-PEG-L and PTX-PEG-ML NPs were determined to be 296, 198 nm; -20, and -19 mV, respectively. Also, their drug encapsulation efficiencies were determined to be 97% and 96%, respectively. It was found that PTX-PEG-ML NPs, compared to PTX-PEG-L NPs, caused a reduction (11%) in the rate of drug release. The cytotoxicity of the drug-loaded NPs was assessed using 3-[4,5-dimethylthiazole-2-yl]-2,5-diphenyltetrazolium bromide (MTT) assay against human ovarian epithelial cancer (A2780CP) cells, and the results demonstrated that PTX-PEG-ML NPs caused higher cytotoxicity (by 14%) compared to PTX-PEG-L NPs (IC_50_: 1.88 ± 0.09 and 2.142 ± 0.1 µM, respectively).

***Conclusion:*** Overall, the results of this study suggest that PTX-PEG-ML NPs could be considered as a therapeutic candidate for the treatment of ovarian cancer.

## Introduction

Since 1970, the elemental-form of magnetic powders have been used in biological and medical science research.^1^ Magnetic nanoparticles (MNPs) are one of the most widely used types of nanomaterials, due to their unique properties and specific functionality compared to other nanostructures. High biocompatibility and low toxicity are some advantages of magnetic iron oxide NPs.^2^

Jiang et al^3^ demonstrated that magnetic iron oxide NPs were toxic at the concentrations of 12.5 to 75 µg/mL on SKOV3/DDP cells.^3^ MNPs, with a magnetic flux and hyperthermia effect, causes a high percentage of cancer cell elimination, where these NPs are kept under the influence of high-frequency alternating magnetic field to generate heat. Researchers have shown that cancer cells are irreversibly destroyed at 42-45℃.^4,5^ The half-life of MNPs can be significantly increased by modifying their surfaces with hydrophilic polymers, such as polyethylene glycol (PEG), causing a long-term and sustained drug release in the body.^6,7^ Cancer is the inconsistency between growth and cell death, resulting in an excessive number of cells.^8^ Ovarian cancer is the fifth leading cause of death from cancer among women (184 799 deaths in 2018) and is the most lethal gynecologic malignancy.^9-11^ Approximately 68% of the cases are diagnosed at the late stage, leading to a reduction in their survival rates compared to those diagnosed at an earlier stage.^12^ Therefore, in most cases, they are diagnosed at a late stage, which may not be treated, leading to undesirable prognosis.^13^ The five year survival time for patients diagnosed with ovarian cancer is approximately 50%.^14^ Debulking surgery is the first-line treatment in patients with ovarian cancer. At the next step, they receive a chemotherapy regimen, including a platinum-based drug (carboplatin or cisplatin) and a taxane (paclitaxel [PTX] or docetaxel).^15^ Although initial responsiveness to chemotherapy is observed, most of the patients, with advanced ovarian cancer, relapse with the resistant disease. Therefore, the development of more efficient strategies for treating the disease is highly recommended.^15^ In this regard, nanotechnology provides efficient tools, such as NPs, for cancer treatment.^16,17^ NPs can increase the therapeutic effects of drugs and simultaneously decrease their side effects.^7,18^ Liposomes are one of these NPs used for cancer treatment.^16^

Liposomes are a vesicular NP and constructed from concentric lipid bilayers.^19^ From the composition point of view, they are identical to the cell membrane.^20^ Owing to their unique structure, lipid bilayers of liposomes can be loaded with lipophilic and amphiphilic molecules, while the polar liposomal core can be incorporated with hydrophilic molecules.^21^ Liposomes, used in biomedical fields, demonstrate various advantages, including high drug loading capacity (LC), easy synthesis method in a size-controlled manner, controlled drug release, and biocompatibility.^22^

Superparamagnetic iron oxide NPs (SPIONs) have received increasing attention for biomedical use.^23,24^ These NPs can be selectively accumulated in the target tissues or organs owing to their tropism to host cells, biophysical nature, and low toxicity.^25^

The combination of liposomes and MNPs (e.g., Fe_3_O_4_) is still an experimental strategy to design an innovative generation of multifunctional drug delivery systems.^26-28^ This combination results in magnetoliposomes, which are promising nanocarriers for drug delivery to specific tissues and organs, avoiding the side effects of current therapies.^29^

PTX is one of the routine chemotherapeutic agents with high therapeutic effects for ovarian cancer therapy.^15^ Although PTX is one of the most promising chemotherapeutics and the first microtubule-stabilizing agent, it has several limitations, resulting in a decrease in its efficacy. It is a poor aqueous soluble drug and, for this reason, has low bioavailability. To solve this issue, the commercial form of the drug contains a high amount of Cremophor® EL and dehydrated alcohol in 1:1 v/v. However, Cremophor® EL is toxic, and its application is associated with severe side effects, such as hypersensitivity, myelosuppression, neurotoxicity, and nephrotoxicity.^30^

This study aimed to synthesize PTX-loaded PEGylated magnetic liposome NPs (PTX-PEG-ML NPs) and evaluate its cytotoxicity compared to PTX-loaded PEGylated liposome NPs (PTX-PEG-L NPs), and the standard drug against ovarian cancer cells *in vitro*. For this purpose, the nanoformulations were characterized in terms of size, zeta potential, drug encapsulation efficiency (EE), drug LC, and kinetics of drug release using dynamic light scattering, dialysis membrane, and spectrophotometry methods. Then, the cytotoxicity effects of the formulations against ovarian cancer cells were assessed *in vitro* using 3-[4,5-dimethylthiazole-2-yl]-2,5-diphenyltetrazolium bromide (MTT) method.

## Materials and Methods

### Materials 

Cholesterol, PTX, FeCl_3,_ FeCl_2_, phosphate buffer saline (PBS), fetal bovine serum (FBS), Roswell Park Memorial Institute (RPMI) 1640 Medium, penicillin-streptomycin, trypsin, ethylenediaminetetraacetic acid (EDTA),tetramethylammonium hydroxide, and dialysis membrane (6 kDa cut off) were purchased from Merck (Kenilworth, NJ, USA). Soybean lecithin and PEG3350 were purchased from Acros Organics (Geel, Belgium) and Kimyagaran Emrooz Co., (Arak, Iran), respectively. Ethanol (96% v/v) was purchased from Mojallali Chemical Laboratories (Tehran, Iran). A2870CP cell line was prepared from the National Cell Bank (NCBI), Pasteur Institute of Iran, Tehran, Iran.

### Synthesis of iron oxide nanoparticles

Iron oxide NPs were synthesized using two salts (FeCl_3_ and FeCl_2_) and the chemical co-precipitation method with ammonia reduction.^31^ Briefly, an aqueous mixture of FeCl_3_ (8 mL, 1 M) and FeCl₂ (2 mL, 2 M, in 2 M HC1) was added to the NH_3_ solution (100 mL, 0.7 M) to obtain a gelatinous precipitate. The precipitate was then isolated from the solution by centrifugation (1000 RPM, 30 min). The resulting precipitate was treated with 1 M tetramethylammonium hydroxide (N(CH_3_)_4_^+^ OH^−^), and iron oxide NPs were produced. The particles were then stored at 4℃ until the subsequent studies.

### Nanoparticles preparation

PTX-PEG-ML and PTX-PEG-L NPs were prepared using the reverse-phase evaporation method.^20^ For this purpose, 120 mg of lecithin, 45 mg of cholesterol, and 27 mg PEG 2000 (at the molar ratio of 55:40:5) were added into 100 mL of 96% (v/v) ethanol and stirred (200 RPM, 1 h, room temperature). The solvent was evaporated using a rotary evaporator (Heidolph Co., Schwabach, Germany) to obtain a thin yellow layer. Next, 20 mg of PTX and 10 mg of Fe_3_O_4_ NPs were added into the thin layer, and 10 mL of PBS was added into the resulting mixture and stirred (200 RPM, 4 h, room temperature). The mixture was then homogenized (13 000 ×g, 5 min) and sonicated for 5 min (Bandelin Sonorex Digitec, Berlin, Germany; 60 Hz) to produce the PTX-PEG-ML NPs. PTX-PEG-L NPs were synthesized using the same method without adding Fe_3_O_4_ NPs.

### Characterization of nanoparticles

The size and zeta potentials of PTX-PEG-ML and PTX-PEG-L NPs were determined using Zetasizer (Malvern Instruments, Malvern, UK). For this purpose, 0.5 mg/mL of PTX-PEG-ML and PTX-PEG-L NPs were prepared in PBS separately and introduced to the instrument. Also, the NPs were morphologically studied using scanning electron microscopy (SEM) method. For this purpose, the suspension of NPs was separately mixed with maltose (1:2 w/w), and the formulations were lyophilized. Next, 1 mg of the powder obtained from each formulation was coated with gold and visualized using the SEM instrument (XL30, Philips, The Netherlands). Optical density (OD) measurements were carried out to determine EE% in both formulations. First, a standard curve of PTX was plotted by preparing various drug concentrations (1.00, 0.50, 0.25, 0.13, 0.063, 0.031 mg/mL) in PBS at 277 nm. To avoid the interference or overlap of other liposome or NP components, free-drug liposomes were used as the baseline. Next, 2 mL of each formulation was centrifuged (14 000 ×g, 4℃, 30 min), and the absorbance of the supernatant was read at 227 nm using a spectrophotometer instrument (Hitachi, Japan). EE% and LC% were then calculated using the following formulae^32^:

$$EE\%=\frac{Initial\;drug\;concentration\;\left(mg\right)-drug\;concentration\;in\;supernatant\;\left(mg\right)}{Initial\;drug\;concentration\;\left(mg\right)}\times 100$$

$$LC\%=\frac{Drug\;concentration\;in\;nanoparticles\;\left(mg\right)}{Drug\;concentration\;in\;nanoparticles\;\left(mg\right)+nanoparticles\;\left(mg\right)}\times 100$$

### Drug release kinetics

Drug release study was performed using the dialysis membrane method.^16^ To evaluate the drug release kinetics, 1 mL of PTX-PEG-ML and PTX-PEG-L NPs were separately transferred into two dialysis bags (cut-off 6 kDa) and immersed in 100 mL of PBS. At the predetermined time intervals (2, 4, 6, 8, 20, 24, 28, 44, and 48 h), 2 mL of the buffer was replaced with 2 mL of the fresh buffer. The absorbance of the obtained samples was measured at 227 nm spectrophotometrically, and the cumulative percentage of the drug release was calculated using the following formula:

$$Drug\;release\;\left(\%\right)=\frac{Released\;drug\;from\;particles}{Total\;drug\;in\;particle}\times 100$$

### Cell line and culture conditions 

A2780CP cells were cultured in RPMI-1640 medium supplemented with 10% FBS and 1% penicillin/streptomycin in a humidified incubator (37℃, 5% CO_2_). The cells were trypsinized to detach from the flask, and trypan blue staining was used to count the cells.

### Cytotoxicity analysis 

MTT assay was used to assess the cytotoxicity of PTX-PEG-ML and PTX-PEG-L NPs.^33^ For this purpose, 100 µL of the cell suspension (10 000 cells) was added to each well of 96-well plate and incubated for 24 h (37℃, 5% CO_2_). The supernatant was removed, the cells were treated with different drug concentrations (0, 1, 2, 4, 8, 16, 32 and 64 µM) of PTX-PEG-ML and PTX-PEG-L NPs, and incubated for 48 h (37℃, 5% CO_2_). Next, 100 μL of MTT solution was added to each well and incubated for 3 h (37℃, 5% CO_2_) to form the insoluble formazan crystals. All the samples underwent absorbance measurements at 540 nm. IC_50_ (the inhibitory concentration that causes a reduction of 50% in cell growth relative to untreated control)^34^ was calculated using Pharm-PCS statistical package software (Springer-Verlag, NY, USA).

### Statistical analysis

All statistical analyses were performed using GraphPad Prism software version 8.00, and statistical differences were analyzed Student’s *t* test. All results were expressed as mean ± standard deviation (SD, n = 3). The differences between the two tests were considered significant when p-value was less than 0.05.

## Results

### Characterization of nanoparticles

**Figure 1:**
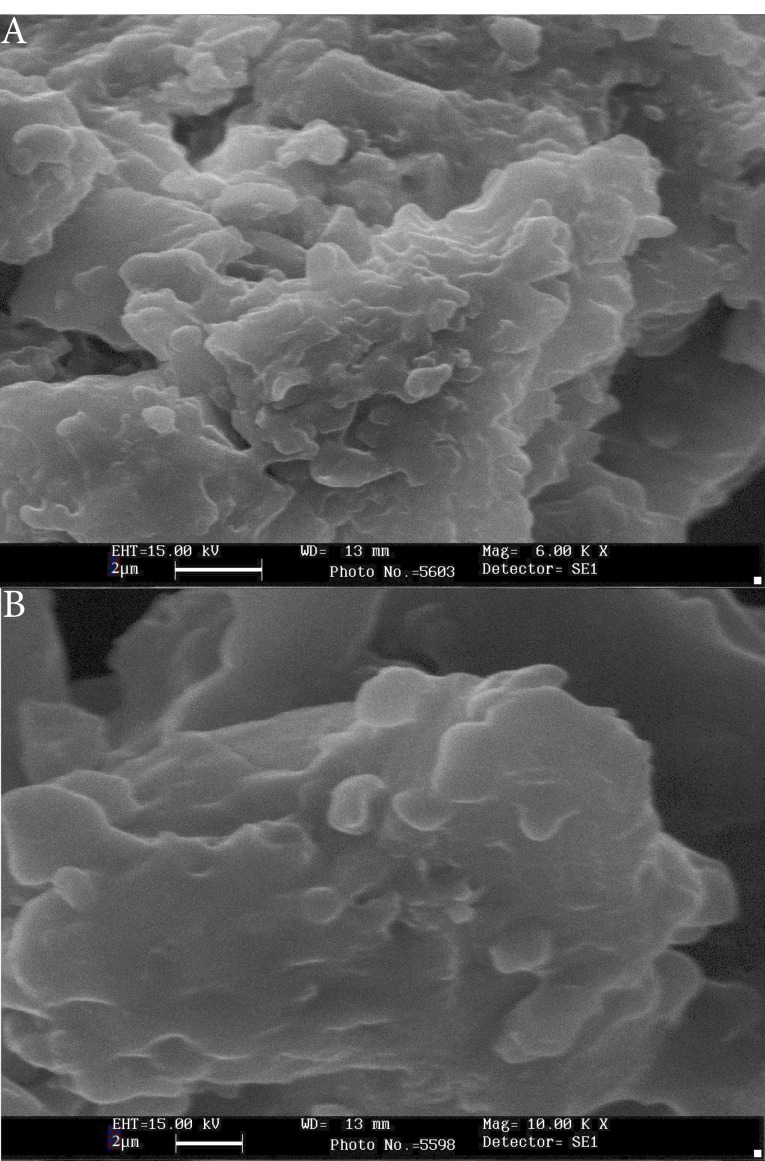
SEM results of (A) PTX-PEG-L (scale bar: 1 µM, Mag: 15000X) and (B) PTX-PEG-ML (scale bar: 1 µM, Mag. 15000X) NPs. As the figure shows, NPs were formed as roundness monodispersed with a smooth surface and without surface fractures or pitting.

According to dynamic light scattering measurements, the mean diameter of PTX-PEG-ML and PTX-PEG-L NPs were 296.1±14 and 197.8±9 nm, respectively. Also, the zeta potentials of these NPs were -19.3±0.9 and -19.8±0.9 mV, respectively. Also, the results of SEM demonstrated that PTX-PEG-ML and PTX-PEG-L NPs were formed as roundness monodispersed (Figure 1).

**Figure 2:**
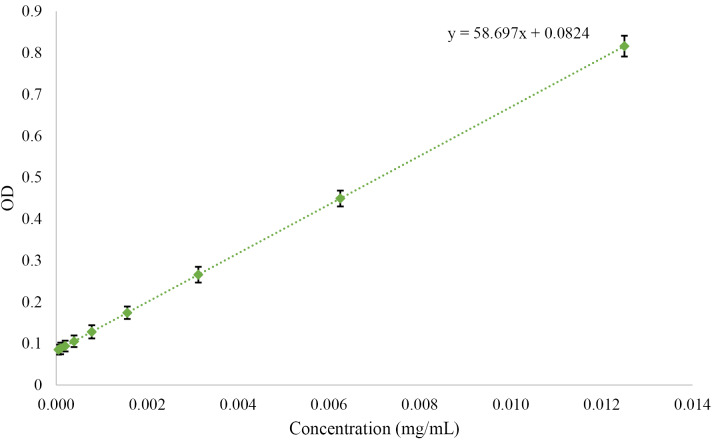
Standard curve of PTX obtained from the absorbance (optical density; OD) of the diluted PTX concentrations.

Furthermore, EE% and LC% were calculated using the standard curve (Figure 2) and the following formula.

$$\text{Optical density }\left(\text{OD}\right)=58/697\text{X }+\;0/0824$$

The results demonstrated that EE% and LC% for PTX-PEG-ML and PTX-PEG-L NPs were 96.46, 97%; and 8.8% and 9.1%, respectively.

### Drug release kinetics

**Figure 3:**
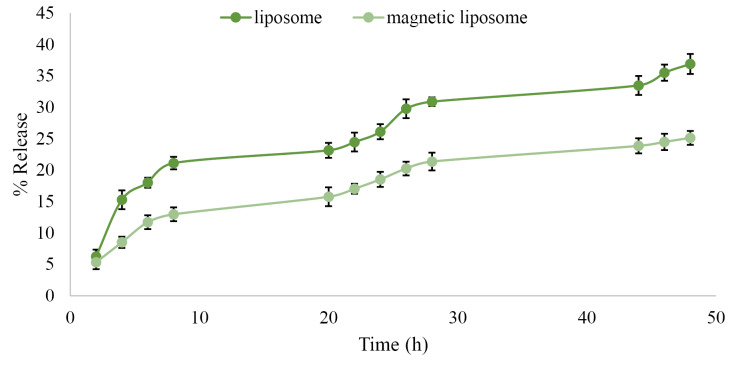
The cumulative PTX release (%) from PTX-PEG-ML and PTX-PEG-ML NPs into PBS in different time intervals (2, 4, 6, 8, 20, 24, 28, 44, and 48 h). Results are presented as mean ± 5% error from three independent experiments.

Figure 3 demonstrated that the drug release from PTX-PEG-L (36.9%) is faster than PTX-PEG-ML NPs (25.1%). The lower rate of the drug release from PTX-PEG-ML compared to PTX-PEG-L NPs could be related to the ability of MNPs to stabilize liposomes and suppress spontaneous leakage of cargo in core water.^35^ According to these results, PTX-PEG-ML NPs preserved the drug in the body for a longer time. Also, as PTX-PEG-ML NPs released the drug at a slower rate, it caused a reduction in the drug toxicity.

### Analysis 

**Figure 4:**
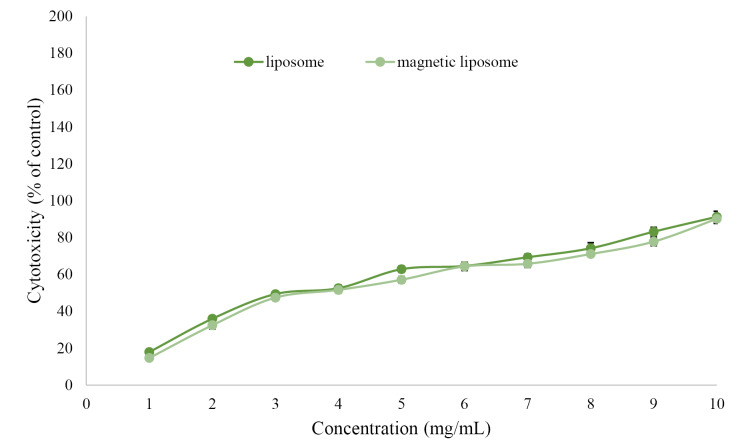
Cell viability of A2780CP, incubated with PTX-PEG-ML and PTX-PEG-L NPs. Data are means of three independent experiments and expressed as means ± SD (*P* ≤ 0.05).

MTT analysis was used to evaluate the cytotoxicity of PTX-PEG-ML and PTX-PEG-L NPs on A2780CP cells. First, the cytotoxicity of MNPs, liposomes, untreated group, and MLs was evaluated, and the results demonstrated that these compounds were non-toxic at the concentrations used in PTX-PEG-ML and PTX-PEG-L NPs structures. The results demonstrated that PTX-PEG-ML compared to PTX-PEG-L NPs caused less cell viability (Figure 4) and higher cytotoxicity effects (by 12%) against the A2780CP cells (IC_50_ = 1.884 ± 0.09 and 2.142 ± 0.1 for PTX-PEG-ML and PTX-PEG-L, respectively).

According to Figure 4, the PTX-PEG-ML NPs demonstrated higher efficiency compared to PTX-PEG-L NPs in increasing the cytotoxicity effects of PTX against A2780CP cells.

## Discussion

Cancer is a collection of diseases recognized by uncontrolled growth and dissemination of abnormal cells.^36^ Ovarian cancer ranks the sixth most common cancer in women.^37^ Approximately 90% of ovarian cancer cases are derived from epithelial cells; however, most of the tumors disseminate into the bloodstream and then extravasate into distant tissue sites.^37^ Currently, the standard treatment regimen for ovarian cancer is surgery and then chemotherapy; however, more than 65% of patients will eventually relapse.^38^ Therefore, the development of more efficient treatment options is highly recommended for the treatment of recurrent or drug-resistant ovarian cancer.

In this regard, nanotechnology provides tools, such as liposomes, to simultaneously and significantly improve the therapeutic effects and decrease the side effects of chemotherapeutics.^18,39^ Liposomes are able to be selectively accumulated in the tumor tissues through the leaky tumor vasculature, avoiding off-target effects of the drug.^40^ Magnetoliposomes have potential applications for diagnosing and treating cancers and evaluating the response to treatment.^41^

In the present study, PEG was incorporated into magnetoliposome. PEG coats the surface of liposomes and improves the aqueous solubility, causing an increase in the blood circulation time of liposomes by decreasing their uptake by macrophages.^42-44^ Increasing the liposome stability and blood half-life causes a higher amount of liposomes to be accumulated into tumor tissues through the leaky vasculature tumors and dysfunctional lymphatic drainage, leading to an increase in the therapeutic effects.^17,33^ Also, this coating helps SPIONs (e.g., Fe_3_O_4_) to be simply encapsulated and stabilized into liposomes.^45,46^

In the present study, PTX-PEG-ML NPs were successfully synthesized and characterized, in terms of size, zeta potential, morphology, EE%, LC%, the kinetics of drug release, and cytotoxicity effects. The results demonstrated that nanoscale particles were synthesized. Also, the size of PTX-PEG-ML NPs was significantly larger than that of PTX-PEG-L NPs (296.1±14 vs. 197.8±9 nm), indicating that MNPs were encapsulated into liposome NPs. Furthermore, the zeta potential of both PTX-PEG-L and PTX-PEG-ML NPs were negative, which was in agreement with the results of Ribeiro et al^47^ and Toro-Cordova et al^48^ studies as the magnetoliposome, synthesized in these studies, also had negative zeta potential (-8 ± 1 and -40.5 ± 0.8 mV in Ribeiro et al^47^ and Toro-Cordova et al^48^ studies, respectively). NPs with the same charge (positive or negative) have proper stability in aqueous solutions with low ionic strength as particles with the same net charge repulse each other, which, in turn, inhibits their aggregation.^7^ Also, the results of morphology evaluation indicated that roundness but not spherical NPs were formed. The nonsphericity of the NPs might result from the harsh condition of lyophilization, which can affect the physical stability of liposomes. Also, EE% for both formulations confirmed that the reverse-phase evaporation method used for the preparation of the liposomes was efficient. Furthermore, the results of the drug release study showed that PTX-PEG-ML compared to PTX-PEG-L NPs had a slower drug release rate, indicating that the incorporation of MNPs into the liposomes was an efficient strategy to decrease the drug release rate, and as a result, prolong the drug present in the environment.

The cytotoxicity effects of the formulations were measured on the ovarian cancer cells using MTT assay. The results demonstrated that the anticancer effects of PTX-PEG-ML compared to PTX-PEG-L NPs increased by 12%, demonstrating that the incorporation of MNPs into the liposomes was an efficient strategy to improve the potency of liposomes for increasing the therapeutic effects of PTX. This resulted from the profile of drug release, in which PTX-PEG-ML compared to PTX-PEG-L NPs had a slower drug release pattern, resulting in an increase in the exposure time of the drug with cells. These results were in agreement with the results of Cruz dos Santos et al^49^ study, where they demonstrated that quercetin-loaded magnetoliposome compared to quercetin-loaded liposome caused a reduction of 16% in the viability of rat glioblastoma C6 cells. Rodrigueset al^50^ also demonstrated that magnetoliposomes could increase the antitumor effects of N-(3-methoxyphenyl) thieno [3,2-b]pyridin-7-amine by 4% (GI_50_ = 5.67 ± 0.62 vs. 5.88 ± 0.86) against human breast cancer MCF-7 cells. GI_50_ is the concentration that causes 50% inhibition of cell growth.^51^ Ribeiro et al^47^ used magnetoliposome for co-delivery of PTX and gemcitabine. They evaluated the cytotoxicity effects of the resulting formulation against human breast cancer MGSO-3 cells. The results demonstrated that PTX gemcitabine-loaded magnetoliposome caused higher cytotoxicity effects (by 27%) compared to PTX-gemcitabine-loaded liposome, after 48 h incubation. The higher potency of PTX-gemcitabine-loaded magnetoliposome compared to PTX-loaded magnetoliposome synthesized in the present study (27 vs. 12%) could be originated in the components used for synthesizing these formulations, the origin of the cells (e.g., ovarian vs. breast cancer), and the protocol used for the evaluation of the cytotoxicity effects (i.e., magnetoliposome for co-delivery of PTX and gemcitabine in Ribeiro et al^47^ study vs. magnetoliposome for PTX delivery in the present study). Toro-Cordova et al,^48^ in another study, synthesized cisplatin-loaded magnetoliposome and evaluated its cytotoxicity effects against human cervical cancer HeLa cells. The results demonstrated that magnetoliposome, compared to the liposome, caused an increase in the cytotoxicity effects of cisplatin by 25%. Overall, the results of the present study demonstrated that the incorporation of MNPs into the liposome NPs is a promising approach to improve the potency of liposome for increasing the therapeutic effects of PTX.

## Conclusion

PTX-PEG-ML and PTX-PEG-L NPs were successfully synthesized using the reverse-phase evaporation method. The results demonstrated nanoscale size particles were synthesized. Both formulations released the loaded PTX in a controlled manner; however, PTX-PEG-ML compared to PTX-PEG-L NPs released the lower amount of the drug, demonstrating its higher potency to preserve the drug into NPs. Therefore, this can enhance the chance of drug delivery to tumor tissue through the leaky tumor vasculature. The results of the cytotoxicity study also demonstrated that PTX-PEG-ML compared to PTX-PEG-L NPs were more efficient in enhancing the cytotoxicity effects of PTX against A2780CP cells. This stems from the slower drug release from PTX-PEG-ML. Overall, the results of the present study suggest that the incorporation of MNPs into liposome NPs is a promising strategy to improve the potency of liposome for increasing the anticancer effects of PTX.

## Ethical Issues

Not applicable.

## Conflict of Interest

The authors declare no conflict of interest.
